# ROS Involves the Fungicidal Actions of Thymol against Spores of *Aspergillus flavus* via the Induction of Nitric Oxide

**DOI:** 10.1371/journal.pone.0155647

**Published:** 2016-05-19

**Authors:** Qingshan Shen, Wei Zhou, Hongbo Li, Liangbin Hu, Haizhen Mo

**Affiliations:** Department of Food Science, Henan Institute of Science and Technology, Xinxiang, China; Fujian Agriculture and Forestry University, CHINA

## Abstract

*Aspergillus flavus* is a well-known pathogenic fungus for both crops and human beings. The acquisition of resistance to azoles by *A*. *flavus* is leading to more failures occurring in the prevention of infection by *A*. *flavus*. In this study, we found that thymol, one of the major chemical constituents of the essential oil of *Monarda punctate*, had efficient fungicidal activity against *A*. *flavus* and led to sporular lysis. Further studies indicated that thymol treatment induced the generation of both ROS and NO in spores, whereas NO accumulation was far later than ROS accumulation in response to thymol. By blocking ROS production with the inhibitors of NADPH oxidase, NO generation was also significantly inhibited in the presence of thymol, which indicated that ROS induced NO generation in *A*. *flavus* in response to thymol treatment. Moreover, the removal of either ROS or NO attenuated lysis and death of spores exposed to thymol. The addition of SNP (exogenous NO donor) eliminated the protective effects of the inhibitors of NADPH oxidase on thymol-induced lysis and death of spores. Taken together, it could be concluded that ROS is involved in spore death induced by thymol via the induction of NO.

## Introduction

*Aspergillus flavus* is a well-known saprotrophic and pathogenic fungus for its colonization of cereal grains, legumes and tree nuts [1], and many strains can produce toxic compounds, especially aflatoxin, which leads to rapid death and chronic outcomes such as hepatocellular carcinoma [2]. In addition, as an opportunistic human and animal pathogen, *A*. *flavus* causes aspergillosis in immunocompromised individuals [3]. In some tropical countries (e.g. India, Sudan, Kuwait, and Iran), *A*. *flavus* is also proved predominantly responsible for fungal rhinosinusitis and fungal eye infections (endopthalmitis and keratitis) [4–6]. To eliminate the adverse effects of *A*. *flavus*, some antifungal agents (e.g. voriconazole) have been developed to treat infections caused by this fungus. However, recent reports have showed clinical failures due to the acquirement of resistance to azoles by *A*. *flavus* [7–9]. Deep perception of mechanisms underlying death of *A*. *flavus* will contribute to the development of new efficient drugs against this pathogen.

Many kinds of essential oils have been showed with antifungal activities, and are obtaining intensive concern including in the control of *A*. *flavus* [10–12]. Thymol, one of major chemical constituents of essential oil in *Monarda punctate*, is capable of affecting the surface electrostatics of cell membrane and membrane integrity and killing fluconazole-resistant *Candida* isolates [13,14]. Experiments *in vitro* indicated that thymol inhibits H(+)-ATPase in the cytoplasmic membrane [15], ergosterol biosynthesis [13], and drug efflux pumps [16]. Through cell-based screen, Darvishi [17] built a new mode of thymol antifungal activity through inhibiting transcription of *EST2* and thus telomerase activity, accelerating telomere shortening, and then increasing the rate of cell senescence and apoptosis. Taken together, thymol as an active small molecule, probably has several targets in the fungal cell. Recent studies show although many antibiotics have different targets in bacteria, the lethal actions are common by the generation of reactive oxygen species (ROS) [18,19]. Thymol can also trigger the eruption of ROS to kill Methicillin-resistant *Staphylococcus aureus* [20]. Whether ROS involves the fungicidal action of thymol remains to be elucidated. In this study we determined the antifungal activity of thymol against *A*. *flavus*, and discovered the involvement of ROS in fungicidal action via inducing nitric oxide (NO).

## Materials and Methods

### Chemicals

Thymol (≥99.0%) was purchased from SIGMA. The probes for ROS and NO detection were obtained from Beyotime. Other chemicals were reagent grade.

### Spore suspension preparation

*A*. *flavus* CGMCC3.2890 was obtained from the China General Microbial culture collection Centre and recovered on the Sabouraud Dextrose (SD) medium containing 4% (w/v) glucose, 1% (w/v) peptone and 1.5% (w/v) agar. The spore suspension was collected by shaking the strain plate slightly with the addition of 0.1% Triton X-100. And spore numbers were counted by using a blood counting chamber under the microscope (Motic, BA210).

### MIC measuring

Spore suspension of *A*. *flavus* was inoculated into the SD liquid medium with final concentration of 2×10^6^ spores per milliliter, and the spore medium was divided into the cells of 96-well plate. Thymol was then added into the wells with the final concentrations of 0, 25, 50, 80, 100, 150 and 200 μg/mL, respectively. The plate was incubated at 30°C for 48h, and the growth of *A*. *flavus* in each well was assessed and compared through observation. And the optical density in each well was detected at 600nm through a microplate reader (Thermol, Varioskan Flash). The MIC (Minimum Inhibitory Concentration) of thymol to *A*. *flavus* was defined as no visible growth in the wells [21].

### Spores viability assay

Spore numbers in wells were counted by using a microscope, and spores viability was analyzed by transferring the spore suspension onto SD agar plates. The spores were washed once with the saline solution before determining their survival. The suspension of spores was serial-diluted and transferred onto SD plates, and then incubated at 30°C for 12 h. The viable spores were calculated by counting the colony numbers on the plate.

### Mycelial biomass assay

Spores suspension was inoculated into 20mL SD liquid media (10^5^ /ml) containing 0, 20, 40, 60, 80 and 100 μg/mL thymol, respectively. All treatments were incubated at 30°C, 150 rpm for 48 h. The dry weight of *A*. *flavus* mycelia were weighed after filtering (Millipore filters; 0.45μm pore size) to remove the medium and drying at 80°C for 24 h.

### Scanning electron microscopy (SEM)

*A*. *flavus* spores suspension (2×10^6^/mL) was exposed to 200 μg/mL thymol at 30°C, 150 rpm for 24 h. Then all samples were centrifuged at 8000 g for 5 min to remove the media, washed with 1mL phosphate buffer saline (PBS, pH 7.4), and then fixed with pre-cooled 2.5% glutaraldehyde (Sigma Chemical, St. Louis, MO, EUA) at 4°C overnight. The fixed spores were washed with PBS, dehydrated with ethanol (from 30% to 100%) and then centrifuged at 8000 g for 5 min to remove ethanol. The pellets were resuspended twice with isoamyl acetate in an interval of 20 min. Finally, 10 μL spores suspension in isoamyl acetate was transferred onto an 8-mm disk and was dried at room temperature. All the treated samples were coated with gold in a metallizer (Shimadzu IC-50, Kyoto, Japan) before they were observed by SEM (SEM Shimadzu SS550, Kyoto, Japan).

### ROS and NO assay

ROS in spores was visualized using DCFH-DA (2’, 7’-dichlorofluorescein diacetate) fluorescent probe described by Foreman [22] and NO in spores was visualized using DAF-FM DA (3-Amino, 4-aminomethyl-2’, 7’-difluorescein, diacetate) fluorescent probe described by Guo [23] through an inverted fluorescence microscope (excitation 488 nm and emission 525 nm) (ECLIPSE, TE2000-S, Nikon). The relative fluorescent density of the fluorescent images was analyzed using Image-Pro Plus 6.0 (Media Cybernetics, Inc.) [24]. All the probes were loaded into the spores by incubation in the cultures containing the probes at 37°C for 30 min before the treatment with thymol. Treated spores were sampled to remove the reagents by washing with the saline solution. Finally, 100 μL spores suspension in saline solution was added into the wells of 96-well plate directly for fluorescent photography. Three kinds of NADPH oxidase inhibitors, imidazole (IMZ, 1.2 mM), pyridine (PY, 10 mM), and diphenylene iodonium (DPI, 10 μM) were applied to prevent the generation of ROS respectively [25,26]. Tungstate (Na_2_WO_4_, 60μM) and N^G^-Monomethyl -L-arginine (_L_-NMMA, 0.4mM) were applied to remove NO as nitrate reductase (NR) inhibitor and nitric oxide synthase (NOS) inhibitor, respectively [27,28]. The 2-(4-carboxy-2-phenyl)-4,4,5,5-tetramethylinidazoline-1-oxyl-3-oxide (cPTIO, 0.2 mM) was applied as NO scavenger [29]. Sodium nitroprusside (SNP, 1 mM) was applied as NO donor. The scavengers and inhibitors of ROS and NO are all dissolved in water except DPI in DMSO.

### Enzymatic activity assay

About 10^8^ spores were collected after the exposure of 200μg/ml thymol and grounded in liquid nitrogen. The obtained powder was homogenized in the ice-cooled 100mM HEPES–KOH buffer (pH 7.5) containing 5mM DTT, 1mM ethylenediamine tetra-acetic acid (EDTA), 10% glycerol, 0.1% Triton X-100, 0.5mM phenylmethylsulfonyl (PMSF), 20μM FAD, 25μM leupeptin, 5μM Na_2_MoO_4_ and 1% polyvinylpyrrolidone (PVP). The homogenate was centrifuged at 13,000 g for 10 min at 4°C. The supernatant was used as the crude extract for the assay of enzyme activities. The activity of NOS and NR was determined with NOS assay kit (Cat No. A1042, Nanjing Jiancheng Bioengineering Institute, Nanjing, China) and NR assay kit (Cat No. A096,Nanjing Jiancheng Bioengineering Institute, Nanjing, China) according to their instructions, respectively.

### Transcript analysis

Spores exposed to 200 μg/ml thymol were sampled and grounded in liquid nitrogen. The total RNA was extracted with RNApure Plant Kit (Cat No. Cw0559, Beijing CoWin Bioscience Co. Ltd., Beijing, China) according to the manufacturer’s instructions. The extracted RNA was treated with DNase I (Takara, Otsu, Japan) at 37°C for 30 min to remove the genomic DNA and then reverse-transcribed to form the first-strand cDNA. The reaction was followed by denaturation at 92°C for 5 min and then cooling to 5°C. The obtained cDNA was then amplified for semi-quantitative analysis. The primers of NOS (forward: 5'-CTGAGCTCCGTGATCTGGTC-3'; reverse: 5'-GAGGCGGTATCCGTACTTCG -3') and NR (forward: 5'-CCCGGTCAAATAGGAGGACG-3'; reverse: 5'-AGTACATATCGCGAGGCTGC-3') were designed by Primer-blast in NCBI (http://www.ncbi.nlm.nih.gov/tools/primer-blast). The 18S rRNA gene was used as the internal standard (forward 5'-ATGGCCGTTCTTAGTTGGTG-3'; reverse: 5'-GTACAAAGGGCAGGGACGTA-3'). The PCR programs included 30 cycles of 95°C for 30 s, annealing at 55°C for 20 s, and extension at 72°C for 1 min. The RNA samples were tested for genomic DNA contamination with the extracted RNA directly as the PCR template prior to cDNA synthesis and under the same PCR conditions. The RT-PCR products were separated on 2% agarose gels and stained with ethidium bromide. All of the RT-PCR reactions were performed at least three times. Densitometric scanning using a computer-assisted image analysis system (IP Lab Gel; Signal Analytics Corp., Vienna, VA, USA) was used to quantify the signal intensity of each band. The relative abundance of the transcripts were obtained by dividing the band intensity of target gene by the band density of the corresponding 18S rRNA.

### Statistical analysis

Each result was presented as the mean ± standard deviation (SD) of at least three replicated measurements. Significant differences between treatments were statistically evaluated by SD and one-way analysis of variance (ANOVA) using SPSS 2.0. The data between two specific different treatments were compared statistically by Student’s t-test. The differences were considered significant at P<0.05.

## Results

### Antifungal activity of thymol against *A*. *flavus*

**Fig 1:**
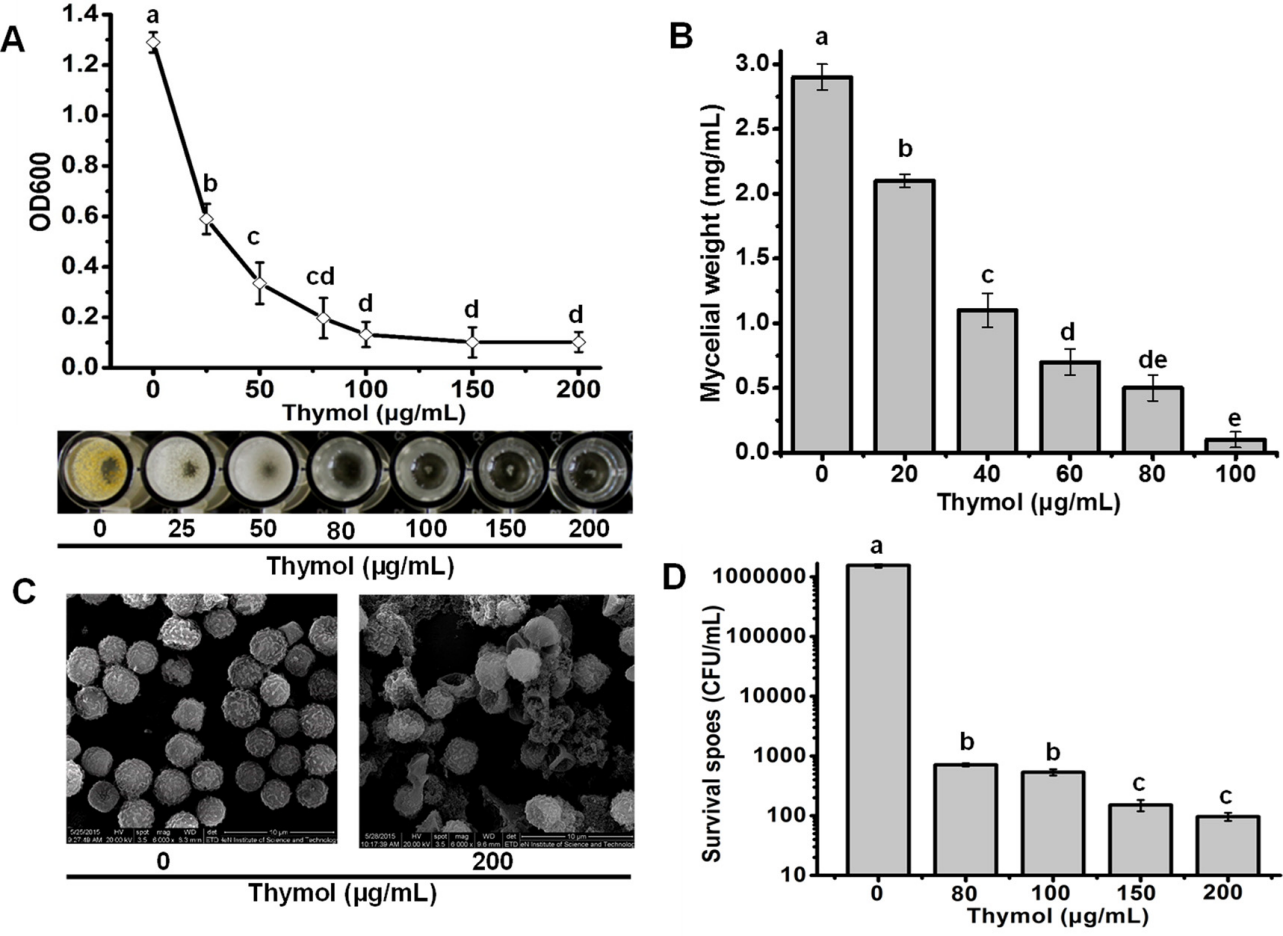
Antifungal effects of thymol on the *A*. *flavus* spores. (A) The spore growth in the wells containing 0, 25, 50, 80, 100, 150 and 200 μg/mL thymol determined by the OD_600_ values and visible observation. (B) Mycelial biomass formations in the presence of thymol at different concentrations. (C) The morphology of spores exposed to 0 and 200 μg/mL thymol under SEM (20.0KV, 6000×). (D) The survival of spores in the wells exposed to more than 80μg/mL thymol. Each data point or bar was indicated as the means of 3 replicates ± standard deviation. Different letter indicate a significant difference between them (P <0.05).

Thymol showed efficient antifungal activity against *A*. *flavus*. The growth of spores decreased in the wells of 96-well plate with higher concentration of thymol. Almost no visible mycelia could be observed when the well containing more than 80 μg/mL of thymol, which was in line with the data of OD_600_ for each well (Fig 1A). The MIC of thymol against spore growth of *A*. *flavus* was defined as 80 μg/mL. Antifungal activity against *A*. *flavus* by thymol also could be found from the mycelial biomass formation (Fig 1B). More thymol led to less mycelial formation, and almost no mycelial ball formation could be visibly observed in the presence of 80μg/mL thymol (Fig 1B). To confirm whether the fungicidal effects or the fungistatic effects was involved in antifungal activity of thymol, we counted the number of spores including all spores and the viable spores remaining in the wells containing more than 80 μg/mL thymol. Seldom spores could be observed through microscopy. Considering the initial number (2×10^6^/ml) of spores, it could be inferred that most of the spores had lysed post the exposure to more than 80μg/mL thymol. It was also supported by the results from the SEM results, indicating that considerable amounts of spores were lysed and much cell debris was produced with the exposure to 200 μg/mL thymol for 24 h (Fig 1C). Through viability assay, it was interesting to find that there were still surviving spores remaining in the wells even containing 200 μg/mL thymol (Fig 1D). However, more than 99.9% cells were killed efficiently by thymol. The survivors did not grow further in the presence of thymol, and seemed to be dormant cells (Fig 1A). Moreover, the number of survivors decreased very slowly as the concentration of thymol ranging from 80 to 200 μg/mL (Fig 1D). This phenomenon was like microbial drug persistence which has been found extensively in which a subpopulation of microorganisms is able to survive antimicrobial treatment without acquiring resistance-conferring genetic changes [30].

### Thymol induced ROS eruption and NO accumulation in spores

**Fig 2:**
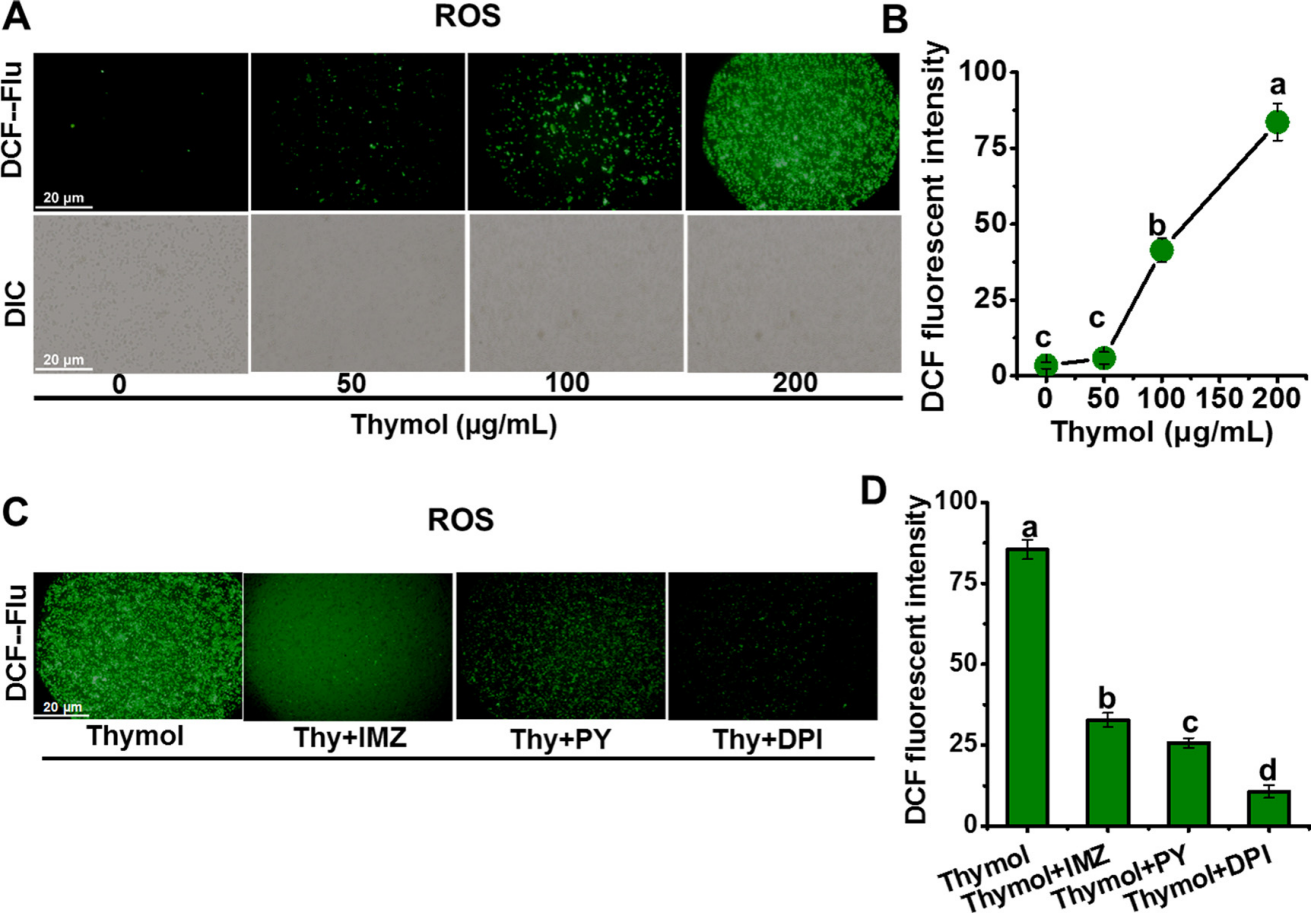
Thymol induced the generation of ROS in spores of *A*. *flavus*. (A, B) The image and relative intensity of DCF fluorescence were obtained when the spores were exposed to thymol (0, 50, 100, 150, and 200 μg/mL, respectively) for 30 min; (C, D) The image and relative intensity of DCF fluorescence were obtained when the spores treated with NADPH oxidase inhibitors (DPI, PY, and IMZ) were exposed to 200μg/mL thymol for 30 min. Each data point or bar was indicated as the means of 3 replicates ± standard deviation. Asterisk indicates that mean values of three replicates are significantly different from the treatment of thymol (P<0.05). Different letter indicate a significant difference between them (P <0.05).

**Fig 3:**
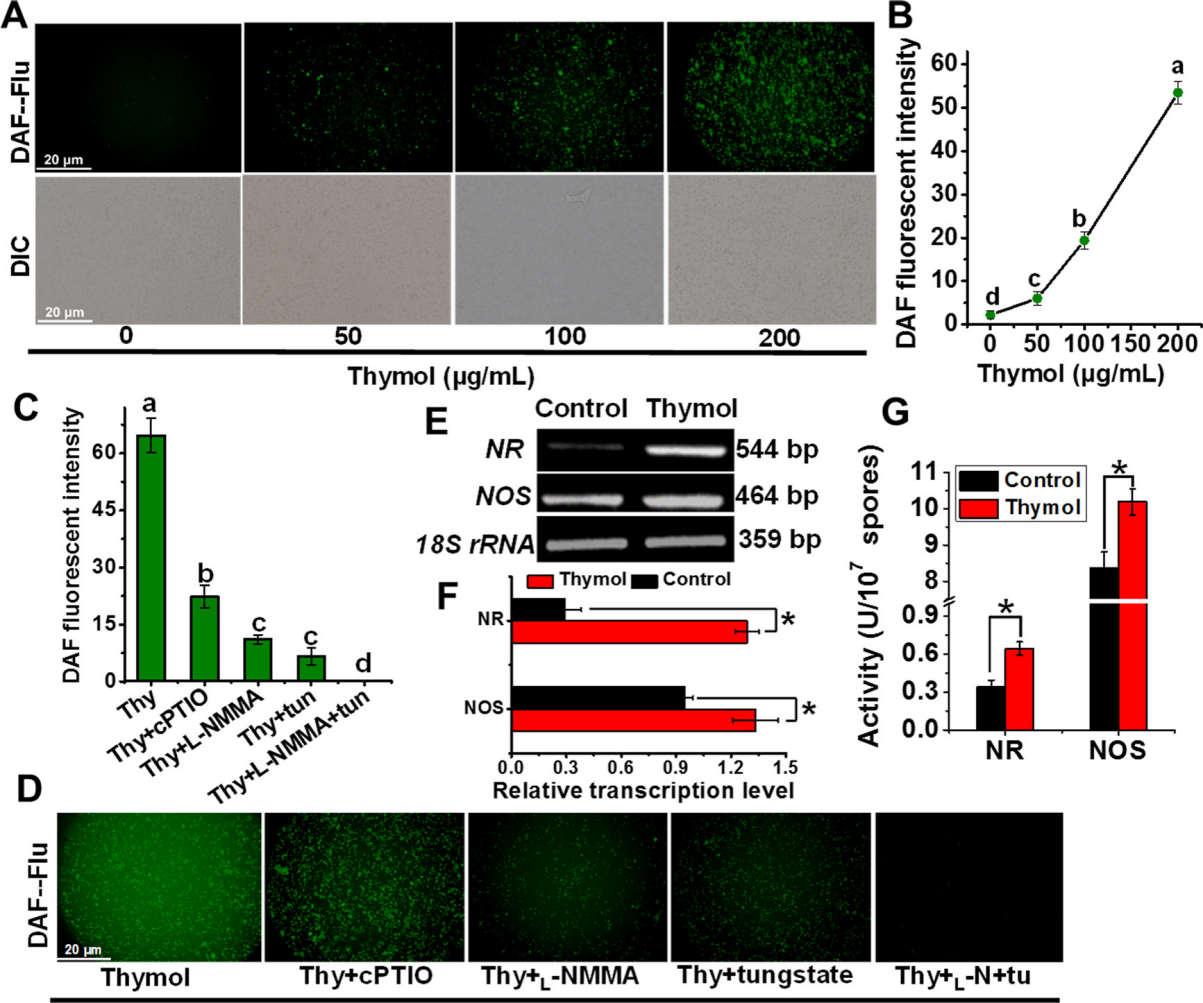
Thymol induced the generation of NO via NR and NOS in spores of *A*. *flavus*. (A, B) The image and relative intensity of DAF fluorescence were obtained when the spores were exposed to thymol (0, 50, 100, 150, and 200 μg/mL, respectively) for 150 min; (C, D) The image and relative intensity of DAF fluorescence were obtained when the spores were exposed to 200μg/mL thymol for 150 min after the treatment with NOS inhibitors (_L_-NMMA), NR inhibitor (tungstate) and NO scavenger (cPTIO), respectively; (E, F) The RT-PCR products in the agarose gel and the relative transcription levels of NR and NOS gene normalized on 18S rRNA level in the spores post the exposure of thymol for 3h; (G) The NR and NOS activities in the spores post the exposure of thymol for 3h. Each data point or bar was indicated as the means of 3 replicates ± standard deviation. Asterisk indicates that mean values of three replicates are significantly different from the treatment of thymol (P<0.05). Different letter indicate a significant difference between them (P <0.05).

Considering the roles of ROS leading to cell death [18,31], we managed to perform *in situ* detection of intracellular ROS level in spores of *A*. *flavus* by using specific fluorescent probe DCFH-DA. At the same time NO generation in vivo also be monitored by using the specific probe DAF-FM DA, because NO often has some associations with ROS involving the signal regulation [29,32]. The results showed both dose-dependent generations of ROS (Fig 2A and 2B) and NO (Fig 3A and 3B) in response to the increasing thymol concentration. The addition of inhibitors of ROS production and NO scavenger (cPTIO) can significantly decrease the fluorescent intensity (Fig 2C and 2D), which confirmed the validity of these fluorescent probes to mark the level of ROS and NO in the spores of *A*. *flavus*. Both _L_-NMMA (NOS inhibitor) and tungstate (NR inhibitor) [27,28] decreased the NO generation (Fig 3C and 3D), and application of their combination led to the removal of most NO, suggesting that NOS and NR contribute to most NO generation in *A*. *flavus* under thymol treatment. Following RT-PCR results confirmed that the transcriptions of *NOS* and *NR* genes were upregulated in response to the treatment of thymol (Fig 3E and 3F), and their enzymatic activities also increased significantly (P<0.05) (Fig 3G). Taken together, it could be concluded that thymol induced both NOS- and NR-dependent NO generation in spores of *A*. *flavus*.

### ROS Induced NO generation in thymol-treated spores

**Fig 4:**
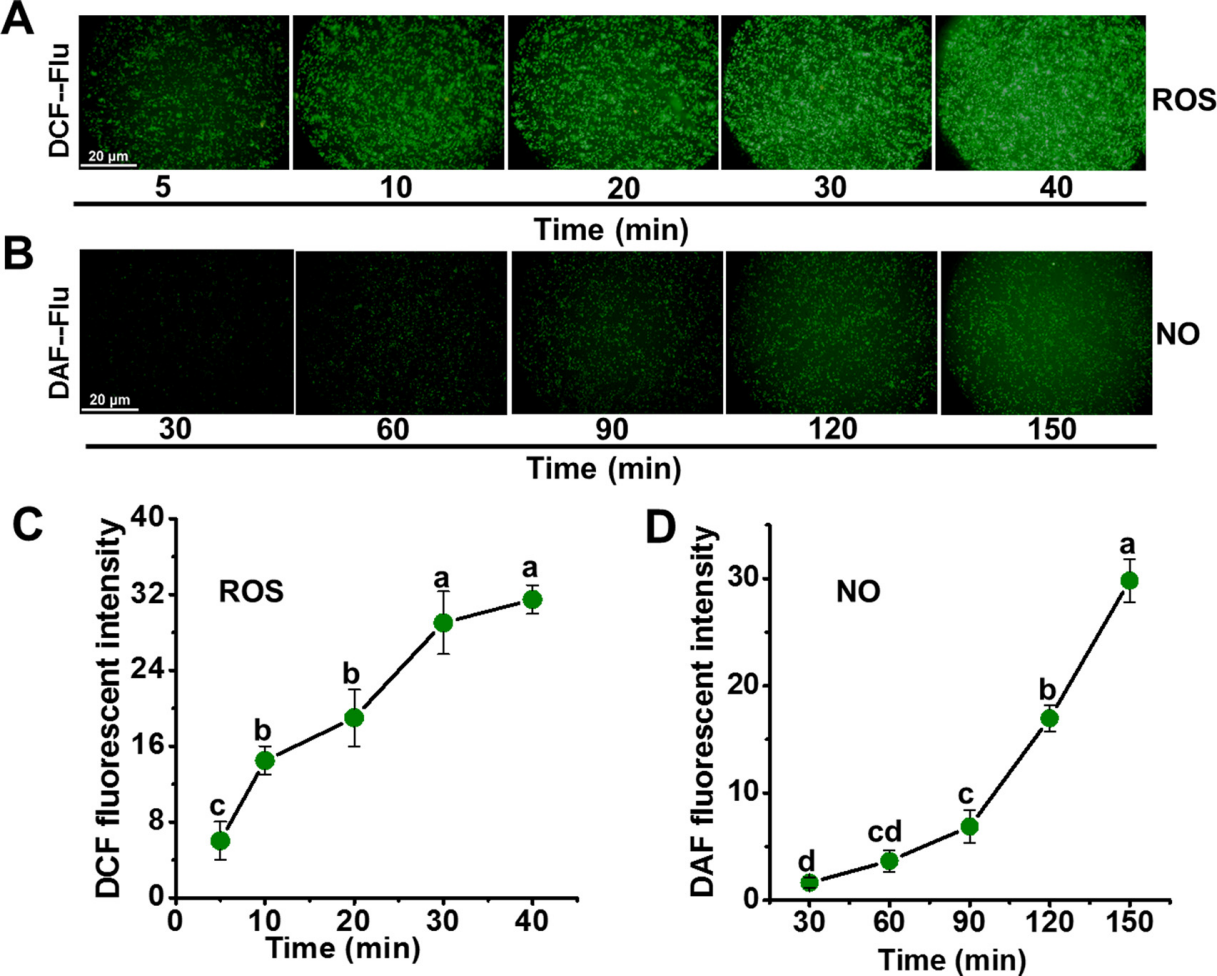
Time course of ROS (A, C) and NO (B, D) generation in spores exposed to thymol. (A) Image of DCF fluorescence was obtained when spores were exposed to 200μg/mL thymol for 0, 10, 20, 30 and 40 min; (B) Image of DAF fluorescence was obtained when spores were exposed to 200μg/mL thymol for 30, 60, 90, 120 and 150 min; (C) Relative intensity of DCF fluorescence; (D) Relative intensity of DAF fluorescence. Each data point was indicated as the means of 3 replicates ± standard deviation.

**Fig 5:**
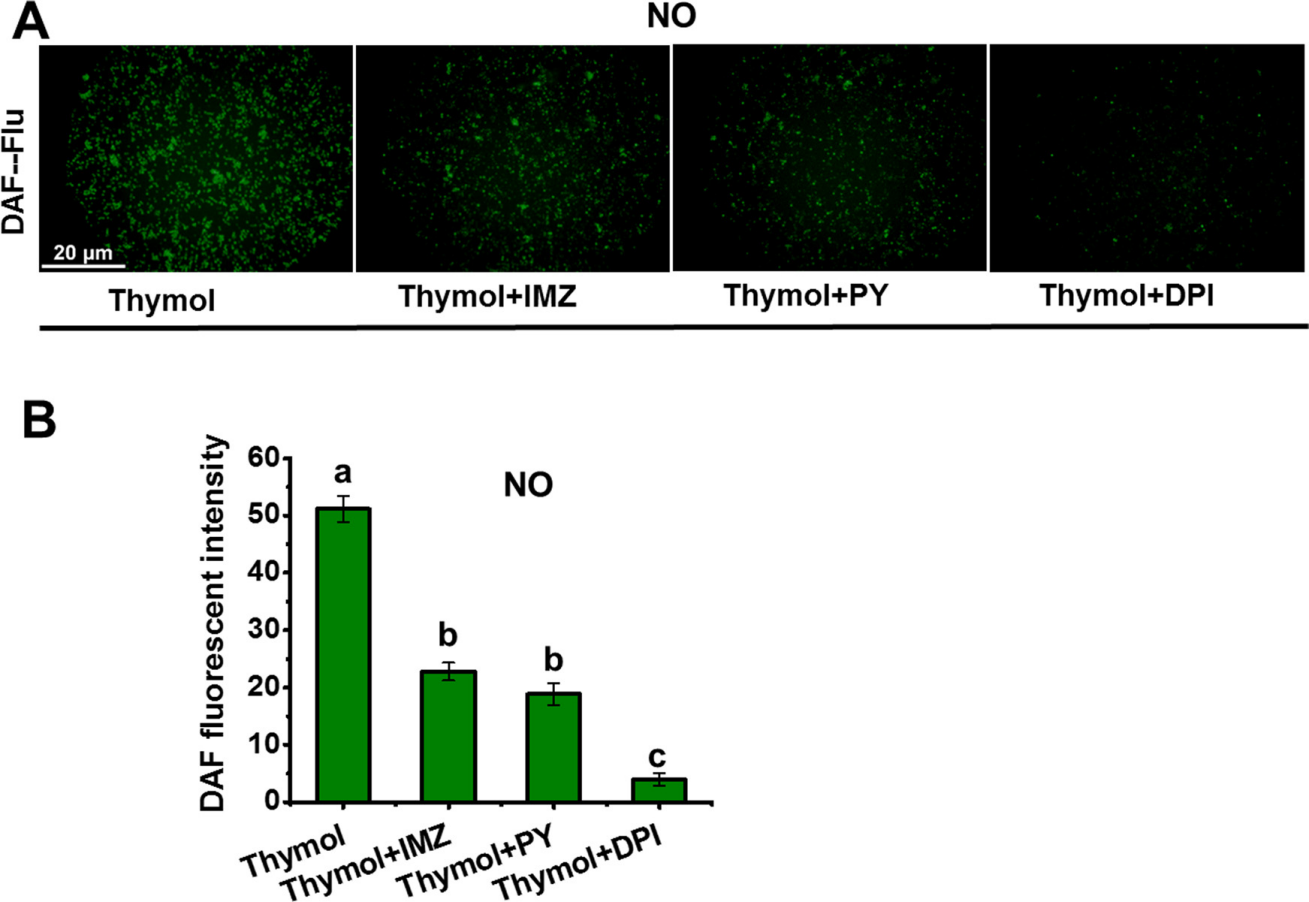
NO generation by blocking the ROS generation in the spores exposed to thymol. (A) The image of DAF fluorescence was obtained when the spores treated with NADPH oxidase inhibitors (DPI, PY, and IMZ) were exposed to 200μg/mL thymol for 150min; (B) Relative intensity of DAF fluorescence. Each data bar was indicated as the means of 3 replicates ± standard deviation. Asterisk indicates that mean values of three replicates are significantly different from the treatment of thymol (P<0.05).

Through the time-course detection, it was found that thymol induced rapid accumulation of ROS in spores (Fig 4A and 4C). Compared to the control, the fluorescence of ROS increased more than 20 times at only 40min post the addition of thymol (Fig 4C). Significant increase in the NO generation mainly occurred at 60 min post the addition of thymol (Fig 4B and 4D), which was later than that of ROS. So a question arose that whether the NO generation was associated with the ROS generation. Thymol-induced NO generation decreased remarkably by blocking the ROS production with the inhibitors of NADPH oxidase (IMZ, PY, and DPI) (Fig 5A and 5B). These results suggested that ROS induced the generation of endogenous NO in *A*. *flavus* in response to thymol treatment.

### ROS Involved in the spore death by thymol via NO

**Fig 6:**
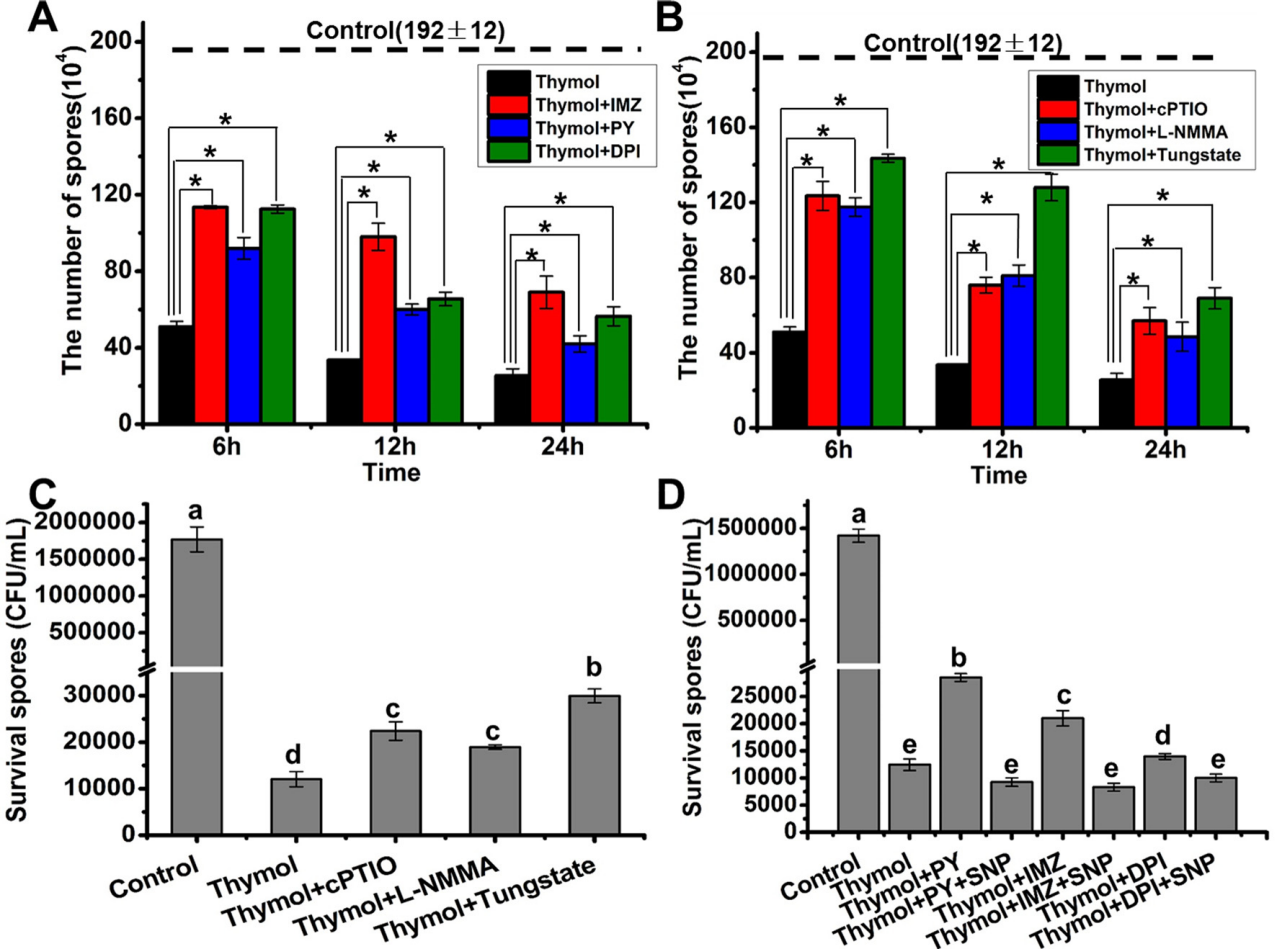
Removal of ROS or NO led to less sporular lysis and death in *A*. *flavus*. (A)The count of remaining spores when they were exposed to 200 μg/mL thymol for 6,12 and 24h after blocking the ROS generation by the addition of NADPH oxidase inhibitors (DPI, PY, and IMZ); (B) The count of remaining spores when they were exposed to 200 μg/mL thymol for 6,12 and 24h after blocking the NO generation by the addition of NOS inhibitors (_L_-NMMA), NR inhibitor (tungstate) and NO scavenger (cPTIO); (C) The survival spores when they were exposed to 200 μg/ml thymol for 12 h after blocking the NO generation by the addition of NOS inhibitors (_L_-NMMA), NR inhibitor (tungstate) and NO scavenger (cPTIO); (D) The survival spores when they were exposed to 200 μg/mL thymol for 12 after blocking the ROS generation by the addition of NADPH oxidase inhibitors (DPI, PY, and IMZ) and then supplying exogenous SNP as the NO donor. The original spore number before the addition of all kinds of drugs is 1.92×10^6^. Each data bar was indicated as the means of 3 replicates ± standard deviation. Asterisk indicates that mean values of three replicates are significantly different between the different treatments (P<0.05)

To determine the roles of the interaction between ROS and NO in spore lysis induced by thymol, the blockers of ROS and NO generation were applied before thymol exposure. The spore number was counted at 6 h, 12 h, and 24 h post the addition of thymol. Based on the existing spore number, it could be found that both the removal of ROS and NO modified the spore lysis by thymol (Fig 6A and 6B). The removal of NO by the addition of NR inhibitor tungstate resulted in the least NO generation in spores (Fig 3D), which allowed the most spores existing from lysis (Fig 6B). Here it should be noted that the spores without lysis did not equal to the surviving spores. After the recovery of these spores on SD plate, most of these spores were dead cells (Fig 6C and 6D). Similar with their protective effects on the spore lysis, both the removal of ROS and NO made more spores survive thymol treatment (Fig 6C and 6D). To confirm the dependence of ROS on NO in the protective effects, we supplied the NO by the addition of exogenous NO donor SNP after blocking the ROS production with the NADPH oxidase inhibitors. The results showed that the supplement of SNP could eliminate the protective roles of ROS removal in the presence of thymol (Fig 6D), whereas KNO_2_ and K_4_Fe(CN)_6_ (byproducts of SNP) had not significant effects (P<0.05) (S1 Fig). This indicated that NO blocked the protective roles of ROS removal. Taken together, it could be concluded that ROS-dependent NO generation was involved in the spore death induced by thymol.

## Discussion

Since slow discovery of new antibiotics has not been competent to resolve continual emergence of resistant strains to antibiotics [33], existing resources have to be reconsidered and re-developed to help overcome this problem. Thymol is one of the most constitutes of thyme, which has historically been applied as a traditional medical purpose against alopecia areata, bronchitis, and cough [34]. Thymol has been registered as food additives in China (FEMA number, 3066) and medical drugs for skin care, dental care and oral hygiene based on its antibacterial activity (www.accessdata.fda.gov/scripts/cdrh/cfdocs/cfcfr/cfrsearch.cfm?fr=310.545). Our results indicated that thymol has the efficient fungicidal activity against *A*. *flavus*, which facilitates it great potential to be developed as drugs for the medical treatment of aspergillosis disease. In addition, some essential oil containing thymol has been used to control the bacterial wilt incidence in tomato [35]. So thymol was supposed to be used on crops to decrease the infection of *A*. *flavus*.

ROS has been revealed to play important roles in cell death among species from bacteria, fungi, plant, and mammals [19,36–39]. Application of some antioxidants can significantly increase the tolerance to the stress by antimicrobials [40]. Through the ROS scavengers, we confirmed the positive roles of ROS in the fungicidal activity of thymol against *A*. *flavus*. Some bacteria generate nitric oxide (NO), which can induce antibiotic tolerance by blocking the Fenton reaction and stimulating antioxidant enzyme action [41]. It is very interesting that NO accumulation also occurs post the eruption of ROS in the *A*. *flavus*. Although NO as a highly reactive molecule has been recognized as an intra-and inter-cellular signaling molecule in animals, plants and microorganisms, it is discovered that fungi are capable of synthesizing NO until the last decade [42]. NO can induce sexual development and affect the morphogenesis in *A*. *nidulans*, which is possible in part via self-inflicted ROS production [43,44]. NO has also proved to involve in the protection of mycelia of edible fungi from heat stress-induced oxidative damage [45]. However, our present data suggested that NO promotes cell death but not protect cell from stresses. Indeed, both NO and ROS are established to be responsible for antimicrobial attack against both bacterial and fungal infection in the mammalian hosts [46]. In plant, endogenous NO is also actually required for cadmium-induced cell death [47], and mediates selenium-induced phytotoxicity by promoting ROS generation in *Brassica rapa* [29]. Out results showed that NO generation was induced by ROS, and involved the fungicidal role of thymol instead of ROS alone. To our knowledge, few reports have mentioned endogenous NO in fungi mediated the cell death in response to the chemical stresses.

How does NO lead to cell death? Many NO-induced effects are mediated by oxidative damage associated with the formation of the potent oxidant peroxynitrite via interaction with superoxide (NO + O_2_^-^ → ^-^ONOO) [32]. But our results showed given the removal of ROS with special scavengers, NO still mediated the spore death of *A*. *flavus* exposed to thymol. Derakhshan and colleagues elucidated [48] that NO signaling in biological systems and the molecular adaptations originated from its ability to selectively target a subset of protein Cys residues by S-nitrosylation. NO signaling cascade leading to cell death has been discovered, and it is initiated by S-nitrosylation of Glyceraldehyde-3-phosphate dehydrogenase (GAPDH) (on Cys150 in the updated sequence), resulting in the specific binding and stabilization of an otherwise labile protein mediator of apoptotic cell death, the E3 ubiquitin ligase, Siah1[49]. By sequence blast and model-template alignment through Swiss-Model, we identified GAPDH in *A*. *flavus* sharing more than 70% sequence identity and high structure similarity with that of in *Rattus norvegicus*. Especially the sequence around Cys residues by S-nitrosylation is the exactly same, which supports GAPDH in *A*. *flavus* might be the target of NO in the presence of thymol. However we could not make sure S-nitrosylation of GAPDH involve the cell death by thymol. After all NO mediated the cell death is dependent on the presence of thymol because the addition of only exogenous NO donor cannot inhibit the spore growth of *A*. *flavus* (S2 Fig). The NO signaling cascade in cell death in *A*. *flavus* induced by thymol remains to be further elucidated, and the effectors in this cascade could be considered as the targets in the new drugs development for aspergillosis or other fungal diseases.

## Supporting Information

S1 Data Dataset for this study. (XLS)

S1 Fig The survival spores when they were exposed to 200 μg/mL thymol for 12h after blocking the ROS generation by the addition of NADPH oxidase inhibitors (DPI, PY, and IMZ). KNO_2_ and K_4_Fe(CN)_6_ are the products released from SNP in aqueous solution. The symbols of “+” and “-” were indicated with or without the treatment, respectively. Each data bar was indicated as the means of 3 replicates ± standard deviation. Asterisk indicates that mean values of three replicates are significantly different between the different treatments (P<0.05)
(TIF)

S2 Fig Effects of SNP on the spore growth of *A*. *flavus*. Fresh spores were inoculated into the wells (spores density: 10^6^/ml) of the 96-well plate containing 200μL SD liquid media with different concentrations of SNP, and then was incubated at 30°C for 24h. The value of OD600 was detected by the microplate reader. Each data bar was indicated as the means of 3 replicates ± standard deviation.
(TIF)
