# Supplementary figures and images for: ROS Involves the Fungicidal Actions of Thymol against Spores of Aspergillus flavus via the Induction of Nitric Oxide
Source: PLoS One. 2016 May 19;11(5):e0155647. doi: 10.1371/journal.pone.0155647 (PMC4872997; doi:10.1371/journal.pone.0155647)

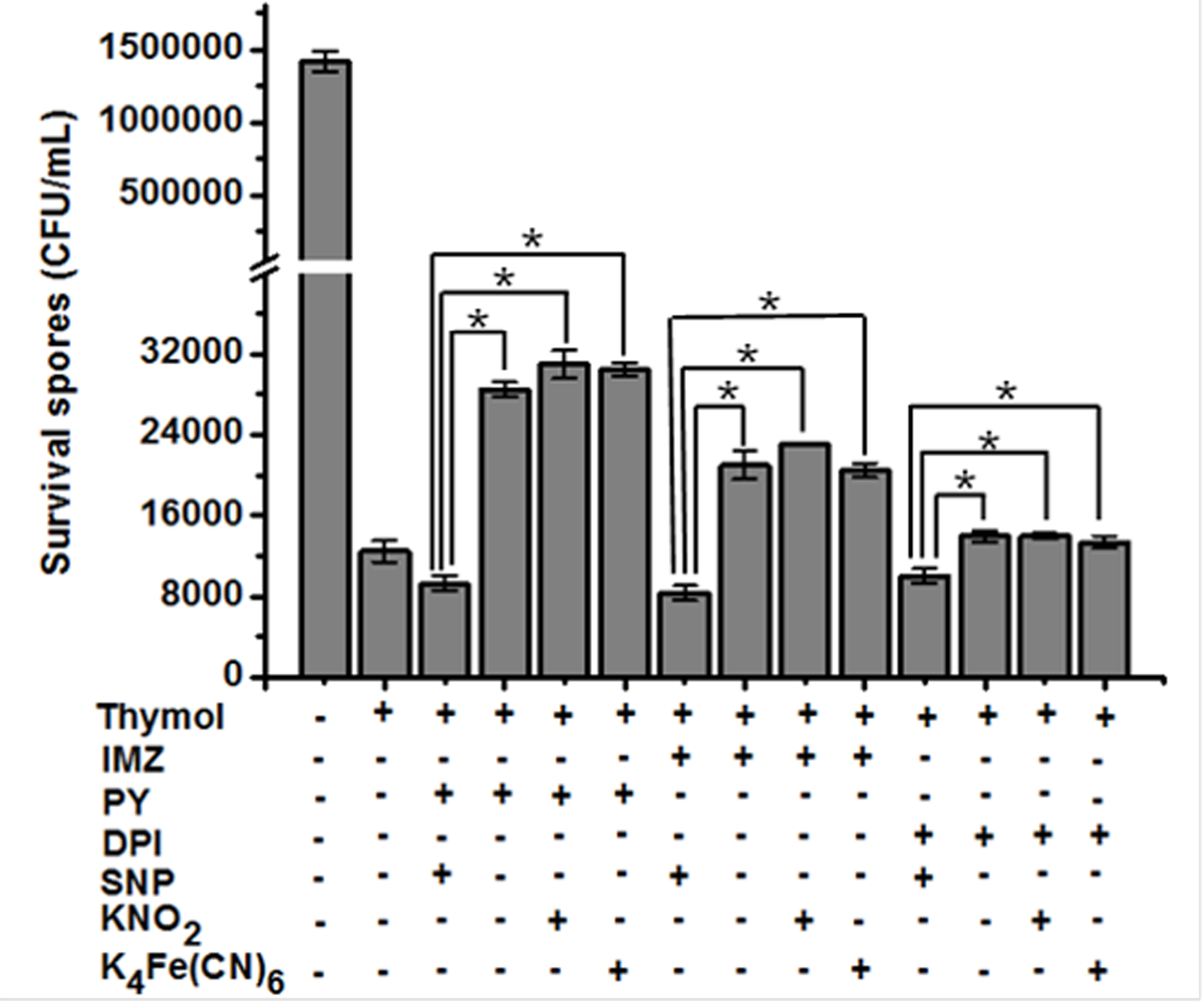

Supplement: S1 Fig — KNO2 and K4Fe(CN)6 are the products released from SNP in aqueous solution. The symbols of “+” and “-” were indicated with or without the treatment, respectively. Each data bar was indicated as the means of 3 replicates ± standard deviation. Asterisk indicates that mean values of three replicates are significantly different between the different treatments (P<0.05) (TIF) [file pone.0155647.s002.tif]

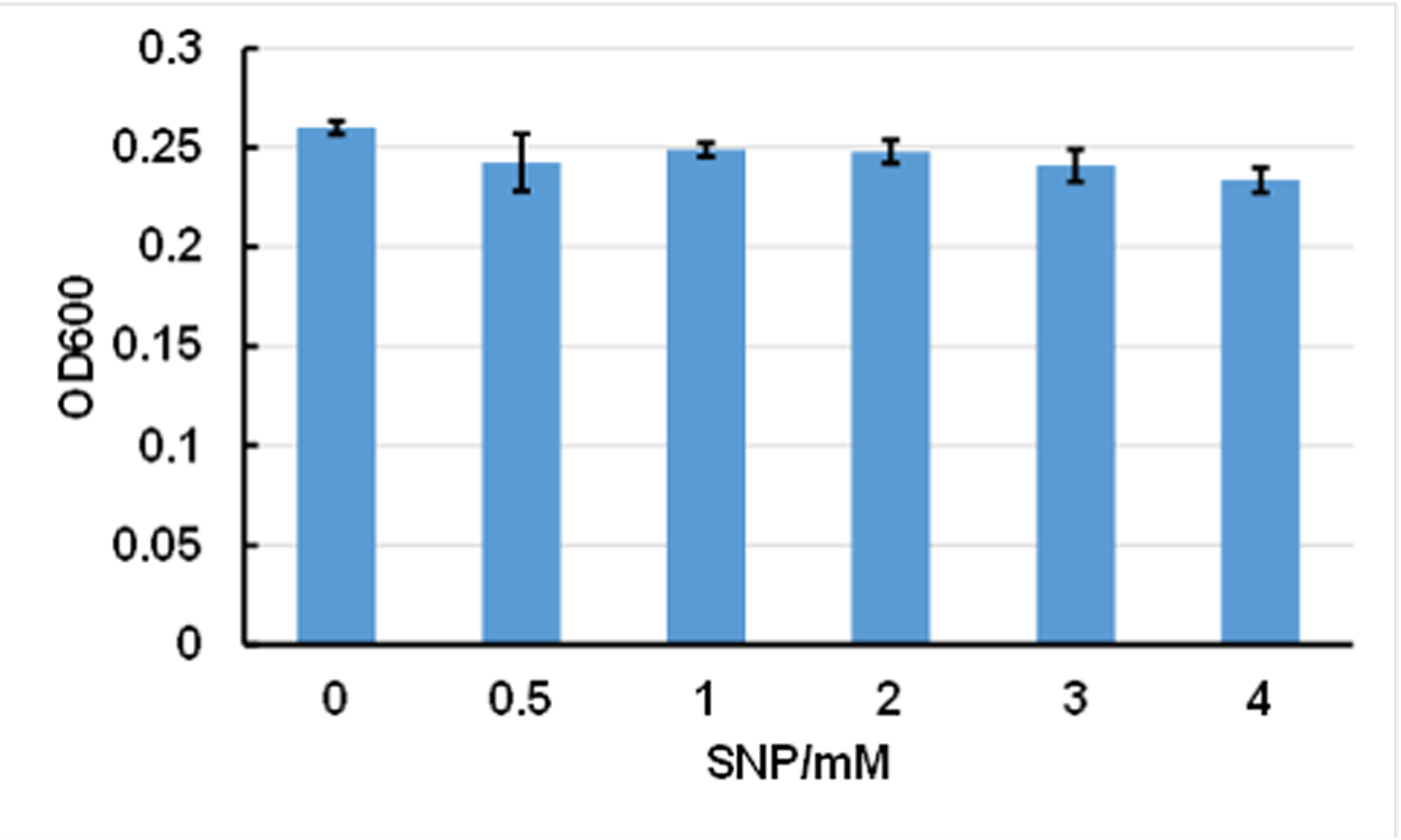

Supplement: S2 Fig — Fresh spores were inoculated into the wells (spores density: 106/ml) of the 96-well plate containing 200μL SD liquid media with different concentrations of SNP, and then was incubated at 30°C for 24h. The value of OD600 was detected by the microplate reader. Each data bar was indicated as the means of 3 replicates ± standard deviation. (TIF) [file pone.0155647.s003.tif]
